# Usage of Synthetic Peptides in Cosmetics for Sensitive Skin

**DOI:** 10.3390/ph14080702

**Published:** 2021-07-21

**Authors:** Diana I. S. P. Resende, Marta Salvador Ferreira, José Manuel Sousa-Lobo, Emília Sousa, Isabel Filipa Almeida

**Affiliations:** 1 CIIMAR–Centro Interdisciplinar de Investigação Marinha e Ambiental, Avenida General Norton de Matos, S/N, 4450-208 Matosinhos, Portugal; dresende@ff.up.pt; 2 Laboratory of Organic and Pharmaceutical Chemistry, Department of Chemical Sciences, Faculty of Pharmacy, University of Porto, 4050-313 Porto, Portugal; 3 Associate Laboratory i4HB-Institute for Health and Bioeconomy, Faculty of Pharmacy, University of Porto, 4050-313 Porto, Portugal; msbferreira@ff.up.pt (M.S.F.); slobo@ff.up.pt (J.M.S.-L.); 4 UCIBIO–Applied Molecular Biosciences Unit, MedTech, Laboratory of Pharmaceutical Technology, Department of Drug Sciences, Faculty of Pharmacy, University of Porto, 4050-313 Porto, Portugal

**Keywords:** peptides, cosmetics, sensitive skin, chemical synthesis

## Abstract

Sensitive skin is characterized by symptoms of discomfort when exposed to environmental factors. Peptides are used in cosmetics for sensitive skin and stand out as active ingredients for their ability to interact with skin cells by multiple mechanisms, high potency at low dosage and the ability to penetrate the stratum corneum. This study aimed to analyze the composition of 88 facial cosmetics for sensitive skin from multinational brands regarding usage of peptides, reviewing their synthetic pathways and the scientific evidence that supports their efficacy. Peptides were found in 17% of the products analyzed, namely: acetyl dipeptide-1 cetyl ester, palmitoyl tripeptide-8, acetyl tetrapeptide-15, palmitoyl tripeptide-5, acetyl hexapeptide-49, palmitoyl tetrapeptide-7 and palmitoyl oligopeptide. Three out of seven peptides have a neurotransmitter-inhibiting mechanism of action, while another three are signal peptides. Only five peptides present evidence supporting their use in sensitive skin, with only one clinical study including volunteers having this condition. Noteworthy, the available data is mostly found in patents and supplier brochures, and not in randomized placebo-controlled studies. Peptides are useful active ingredients in cosmetics for sensitive skin. Knowing their efficacy and synthetic pathways provides meaningful insight for the development of new and more effective ingredients.

## 1. Introduction

Sensitive skin is a condition characterized by the occurrence of symptoms such as tightness, stinging, burning or pruritus, which are triggered by stimuli that do not normally produce unpleasant sensations, such as cold, heat, sun, pollution, cosmetics or moisture [1]. The skin may also present erythema, dryness and desquamation, but these signs are typically absent [2]. Sensitive skin is thought to affect 71% of the general adult population, and the epidemiological studies have also shown that the symptoms are more frequent on the face [3,4]. The causes for this condition are unknown, but genetics, poor mental health, and microbiome imbalance have been proposed as contributing factors [5,6,7]. The pathophysiological mechanisms involved in sensitive skin remain unknown, but three hypotheses have been pointed in scientific literature: increased stratum corneum permeability, an exacerbated immune response and a hyperactivity from the somatosensory and vascular systems [8]. While the two former hypotheses have been questioned and remain poorly understood, there is growing evidence linking sensitive skin to abnormal responses from the somatosensory system. The lower sensitivity threshold in individuals with sensitive skin may be due to a dysfunction in the communication with central nervous system, leading to pain sensations and neurosensory defects, namely the hyperactivation of endothelin receptors and transient receptor potential channels (TRP), which are present in cutaneous nerve fibers such as unmyelinated C fibers and keratinocytes [4,9]. The activation of cutaneous nerve fibers by physical and chemical stimuli, such as heat, low pH solutions, or known irritants such as capsaicin, results in the release of neuropeptides, such as substance P or calcitonin gene related peptide (CGRP), which activate keratinocytes, mast cells, and antigen-presenting cells and T cells nearby, causing a burning pain sensation [10]. A lower density of unmyelinated C-fibers was detected in individuals with sensitive skin, which may be due to degeneration following the contact with the environmental factors, which are thought to be responsible for the occurrence of skin sensitivity. Paradoxically, the lower density of unmyelinated C-fibers may generate hyperreactivity of the existing ones [11]. On the other hand, the inflammatory responses associated to itching sensations are initiated by the activation of transient receptor potential vanilloid type 1 (TRPV1), which is stimulated by heat, capsaicin, and cations, therefore promoting the release of IL-23 by dendritic cells [10]. Individuals with sensitive skin are thought to present an overexpression TRPV1, thus increasing neuronal excitability [12,13]. Overall, these mechanisms may be exacerbated by an impairment in the skin barrier, which fails to protect nerve endings adequately [10].

The synthesis of glutathione in the 1930′s and the isolation of oxytocin in the 1950′s promoted an increase in the research on peptide synthesis, isolation, as well as their chemical, biochemical, and biological characterization [14,15]. After the surge of conformational/topographic-biological activity relationships, which allowed to determine the affinity and specificity for target receptors, peptide leads emerged, offering several advantages over small molecules (increased specificity) and antibodies (small size) [15,16]. Peptide ligands may act as agonists or antagonists at cell receptors and acceptors modulating cell function and animal behavior. This area encompasses approximately 50% of current drugs, and it is likely to keep evolving in the future. In the cosmetic industry, peptides have been used since the late 1980s, with growing notoriety during the first decade of the XXI century [17,18,19]. Peptides used in cosmetic products present a molecular weight lower than 500 Da and hydrophilic properties, thus achieving a moderate penetration through the stratum corneum [20]. Focusing on this challenge, chemical modifications such as esterification with alkyl chains, are usually required. Peptide leads typically derived from three sources: isolated from nature (also known as bioactive peptides); from chemical libraries, or by genetic/recombinant libraries [16]. According to Gorouhi and Maibach, peptides used in cosmetics may be classified as enzyme inhibitory, carrier, neurotransmitter-inhibitory, and signal peptides [17]. Neurotransmitter-inhibitory peptides are able to mimic amino acid sequences involved in neuron excitability, thus modulating the nervous response, while signal peptides stimulate cells’ activity and growth [21]. Accordingly, these peptides may be useful for modulating the neurogenic symptoms associated with sensitive skin, as well as the synthesis of pro-inflammatory cytokines.

We have previously characterized the trends in the use of peptides in anti-aging cosmetics [22]. As the usage of these ingredients in the sensitive skin care segment remains unknown, the present study aims to fill this gap.

## 2. Materials and Methods

### 2.1. Data Collection

The composition of a pool of skin care facial cosmetic products from multinational manufacturers, marketed in Portuguese parapharmacies and pharmacies was collected in 2019, in order to access the most used active ingredients for sensitive skin. Skin care products were included in the study if they exhibited in the label one of the following expressions: “sensitive skin” OR “reactive skin” OR “intolerant skin”. All the information available in the product’s label was collected, along with the information available on the manufacturers’ websites.

### 2.2. Data Analysis

The products ingredient lists were analyzed by visual inspection in order to find peptides, and they were listed according to the International Nomenclature of Cosmetic Ingredients (INCI). Data were analyzed with respect to the following parameters:

#### 2.2.1. Peptides Usage Frequency

The relative amount of cosmetic products for sensitive skin containing peptides were evaluated and expressed in percentage.

#### 2.2.2. Top Peptides for Sensitive Skin

The peptides were identified from INCI lists and ranked in descending order of occurrence to disclose the top.

#### 2.2.3. Scientific Evidence Supporting the Efficacy in Sensitive Skin Care

The efficacy data of each peptide were searched on the on-line databases PubMed, Scopus, Cochrane, KOSMET, and SciFinder. Due to the lack of studies regarding the applicability of active ingredients in cosmetics for sensitive skin, a broader search was performed, using the keywords (“INCI name” OR “synonyms”, when applicable).

## 3. Results and Discussion

Following these criteria, 88 skin care facial products were selected from 19 multinational brands. Fifteen cosmetic products contained one or more peptides in their composition, making up about 17% of products analyzed. Noteworthy, only two products contained more than one peptide in their composition.

### 3.1. Top Ingredients for Sensitive Skin

**Figure 1:**
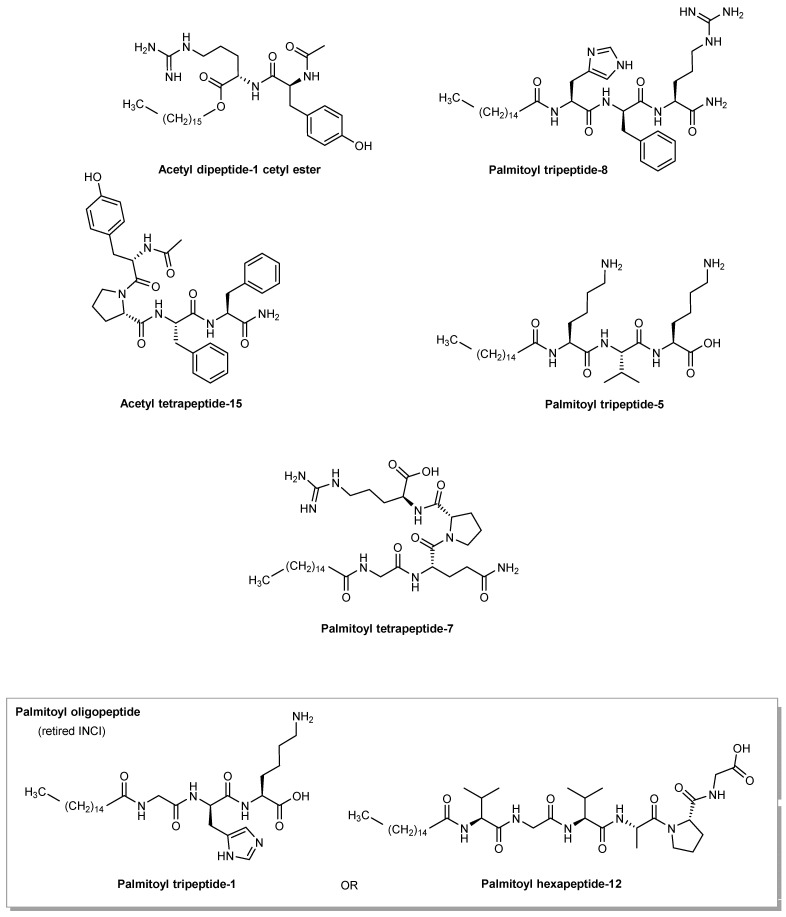
Structures of peptides found at INCI lists from cosmetic products for sensitive skin.

The peptides were identified (Figure 1) and ranked in descending order according to their relative usage (Table 1).

Overall, acetyl dipeptide-1 cetyl ester was the most used ingredient in cosmetic products for sensitive skin, being present in more than 5% of all products. Palmitoyl tripeptide-8 achieved the second place, followed by acetyl tetrapeptide-15 and palmitoyl tripeptide-5. Acetyl hexapeptide-49, palmitoyl tetrapeptide-7, and palmitoyl oligopeptide were only found in the composition of one cosmetic product.

### 3.2. Scientific Evidence Supporting the Efficacy in Sensitive Skin Care

**Figure 2:**
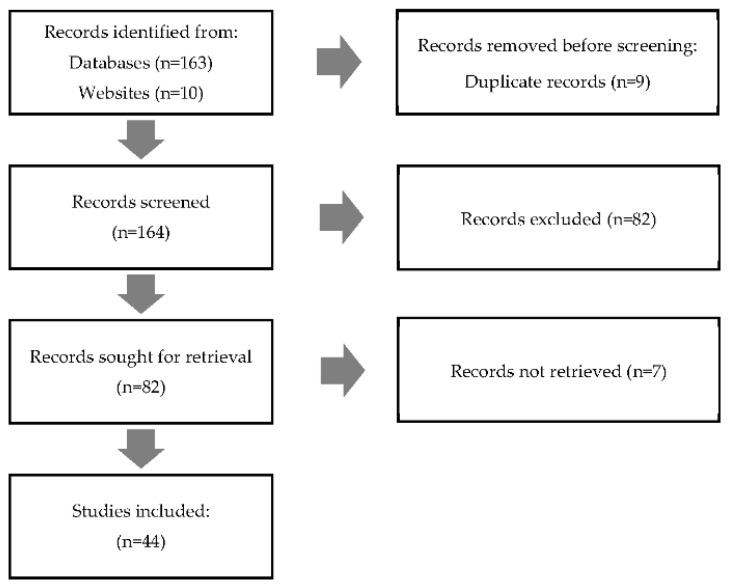
Flow chart of selected articles according to four different parts of the search process: identification, screening, eligibility, and inclusion.

The search results are summarized below (Figure 2):

#### 3.2.1. Acetyl Dipeptide-1 Cetyl Ester

**Scheme 1:**
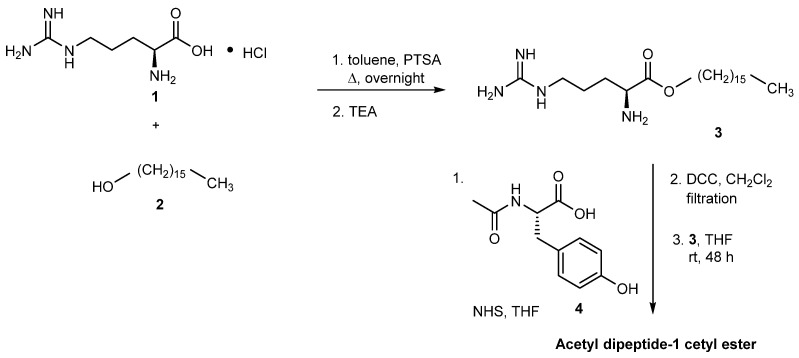
Synthesis of acetyl dipeptide-1 cetyl ester. PTSA: *p*-toluenesulfonic acid; TEA: triethylamine; NHS: *N*-hydroxysuccinimide; THF: tetrahydrofuran; DCC: *N*,*N*’-dicyclohexylcarbodiimide; rt: room temperature; h: hours. Adapted from [23].

Acetyl dipeptide-1 cetyl ester is the INCI name for the peptide *N*-acetyl-L-tyrosyl-L-arginine hexadecyl ester (Figure 1). This compound was based on the bioactive dipeptide Tyr-Arg, for its alleviating and decontracting properties of muscle fibers, and is obtained by chemical synthesis, through initial esterification of L-arginine×HCl (**1**) with palmitol (**2**) [23] to give hexadecyl ether of L-arginine (**3**) (Scheme 1). Activation of *N*-acetyl-L-tyrosine (**4**) with *N*-hydroxysuccinimide (NHS) and coupling with L-arginine (**3**) allows to obtain the acetyl dipeptide-1 cetyl ester [23].

Acetyl dipeptide-1 cetyl ester promotes the pro-opiomelanocortin (POMC) gene expression. POMC incurs post-translational processing and originates the biologically active peptides melanocyte-stimulating hormones (MSHs) and adrenocorticotropin (ACTH), involved in melanin synthesis, as well as β-endorphin, which contains met-enkephalin’s peptide sequence, providing an opiate activity [24,25]. α-MSH is able to bind to melanocytes melanocortin receptors, thus inducing melanin synthesis, but it also intervenes in the reduction of the inflammatory response by modulating the nuclear factor κ-β activity (NF-κβ) [26]. Noteworthy, there are α-MSH peptide fragments which do not elicit significant melanogenic activity, such those that are used in palmitoyl tripeptide-8. Furthermore the opioid β-endorphinreduces CGRP release. CGRP is able to activate TRPV1 in multiple cells thus initiating an inflammatory response [27]. Consequently, acetyl dipeptide-1 cetyl ester reduces the stinging sensation and inflammation resulting from the skin exposure to heat, contact with specific substances, such as capsaicin, and mechanical stress.

Furthermore, Khmaladze et al. demonstrated that acetyl dipeptide-1 cetyl ester significantly upregulates the expression of Aquaporin 3 (AQP3), Filaggrin (FLG), caspase 14, and keratin 10 genes, thus contributing to the improvement of the epidermal barrier [28]. Another study showed that acetyl dipeptide-1 cetyl ester is able to significantly reduce PGE_2_ secretion and decrease NFκB signaling in vitro [29]. PGE_2_ has been proposed to be associated with neurogenic inflammation in sensitive skin [2].

The ingredient supplier reports a reduced ability to perceive heating sensations [30]. The efficacy of a cream containing 3% acetyl dipeptide-1 cetyl ester was assessed regarding the interference in the ability of 21 volunteers to discriminate between four distinct levels of heat: warm, hot, very hot, and painful. The heat perception was reduced very significantly for temperatures which provide hot, very hot, and painful sensations. Additionally, the efficacy of the same cream for reducing the unpleasant sensations provoked by sandpaper aggression on one hand, using the other hand as control, was evaluated in a double-blind study including 18 volunteers. The subliminal response to discomfort was measured by a lie detector. The hand in which the cream was applied revealed greater comfort after the sandpaper aggression. Due to the above-mentioned effects, the supplier concludes acetyl dipeptide-1 cetyl ester is expected to reduce some of the unpleasant sensations of sensitive skin associated with hyperactivity of the somatosensory system.

Schoelermann et al. compared the ability of a cosmetic product containing acetyl dipeptide-1 cetyl ester with another product with 4-*t*-butylcyclohexanol for inhibiting capsaicin-induced stinging in a clinical study including 31 volunteers with sensitive to very sensitive skin. Volunteers’ self-perception stinging/burning sensations and photographs of signs of skin inflammation were used for performing the evaluations. The authors concluded that the product containing 4-*t*-butylcyclohexanol presented a greater efficacy by significantly reducing neuronal activation, compared to the one with acetyl dipeptide-1 cetyl ester, which had no significant effect [31]. However, this study alone does not allow to conclude that 4-*t*-butylcyclohexanol is more efficacious than acetyl dipeptide-1 cetyl ester due to differences in the cosmetic bases containing each active ingredient, which could also interfere with study results.

Moreover, there are several manufacturers who invested in the registration of patents of cosmetic products for sensitive skin that include acetyl dipeptide-1 cetyl ester, demonstrating that researchers and cosmetic manufacturers recognize the value and usefulness of these ingredients for future applications [32,33,34,35,36].

#### 3.2.2. Palmitoyl Tripeptide-8

**Scheme 2:**
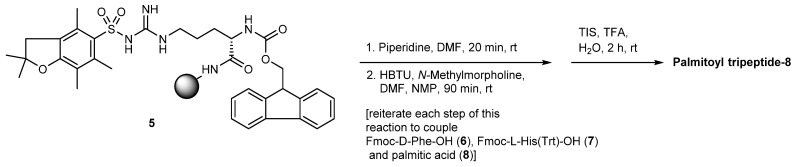
Solid-phase synthesis of palmitoyl tripeptide-8. HBTU: *N,N*,*N*′,*N*′-tetramethyl-*O*-(1*H*-benzotriazol-1-yl)uronium hexafluorophosphate; DMF: *N,N*-dimethylformamide; NMP: *N*-methyl-2-pyrrolidone; TIS: triisopropylsilane; TFA: trifluoroacetic acid. Adapted from [37].

Palmitoyl tripeptide-8 is a synthetic peptide ester based on a α-melanocyte stimulating hormone (α-MSH), originating from POMC, and it is composed by the sequence *N*-(1-oxohexadecyl)-L-histidyl-D-phenylalanyl-L-argininamide (Figure 1). This peptide can be obtained via a solid-phase peptide synthesis using the fluorenylmethyloxycarbonyl (Fmoc) strategy on an ACT496S2 automated synthesizer with PS-Rink amide (RAM) resin (Scheme 2) [37]. The deprotection and coupling steps are carried out until the desired sequences are synthesized. Final side-chain deprotection and cleavage from the resin with a cleavage cocktail (trifluoroacetic acid/water/triisopropylsilane), affords palmitoyl tripeptide-8 [37].

The ingredient supplier performed several efficacy tests. In in vitro models, palmitoyl tripeptide-8 showed the ability to significantly inhibit IL-8 production up to 32% in UVB-irradiated keratinocytes, which was comparable to α-MSH, and also in IL-1 stimulated fibroblasts, reaching 64% inhibition which is greater that the achieved by α-MSH [38]. In skin explants exposed to substance P, palmitoyl tripeptide-8 significantly reduced the number of dilated capillaries and the size of dilated vessels up to 30% and 51%, respectively. Edema was also reduced by 60% due to palmitoyl tripeptide-8 [26]. The supplier also performed two clinical studies. In one study, eight individuals with no reported skin conditions applied both the control and the test formula containing palmitoyl tripeptide-8, three times a day, on separate areas of the volar side of the forearm, for eight days. After this period, single patches containing 250 µL of an aqueous 0.5% sodium dodecyl sulfate (SDS) solution were applied for 24 h, following their removal and a 24 h resting period. Then, the test areas were photographed using a video microscope and their temperature was measured using Thermovision, a temperature measurement device through infrared camera. The supplier reported that photographs demonstrated a redness reduction when palmitoyl tripeptide-8 was applied to the skin, although no quantitative measure was presented. In areas where palmitoyl tripeptide-8 was applied, a significant reduction in the skin temperature after an increase caused by SDS was found. Control results were not statistically significantly different. In another study, which included 13 individuals with no reported skin condition, the same patches containing 250 μL 0.5% SDS solution were applied for 24 h on separate areas of the volar side of the forearm. After this period, the patches were removed and both the test and control formulas were applied three times daily for two days. Again, the test areas were photographed and their temperature was measured using the same equipment. Skin redness decreased in the areas, which the test formula was applied, with no quantitative measurement, and skin temperature increase induced by SDS presented an average 78% reduction, with statistical significance, contrary to control results. Together, these results indicate that palmitoyl tripeptide-8 is able to prevent and soothe an irritative response [26].

There is only one study in the scientific literature, which addresses in vivo the efficacy of a formulation containing palmitoyl tripeptide-8 for the treatment of persistent redness in patients with rosacea who had been successfully treated with topical or oral therapy [39]. Twenty-five patients (23 women and 2 men) were asked to continue using their prior medication, while applying a lotion containing caffeine, zinc gluconate, bisabolol, *Eperua falcata* bark extract, and palmitoyl tripeptide-8 for 8 weeks. Clinical and patients’ assessments for efficacy and tolerability were performed at weeks 4 and 8 using Visia CR device photographs. The evaluation of the product’s efficacy showed a statistically significant improvement in redness, flushing, skin tone, and overall rosacea severity. Skin radiance, texture, and overall appearance also improved. Regarding patient’s tolerance, there was a significant improvement in skin erythema, dryness, edema, and stinging. This finding may be particularly relevant for patients with sensitive skin, who also present this symptom. However, the presence of other active ingredients in the product composition and the lack of control do not allow to draw conclusions regarding palmitoyl tripeptide-8′s efficacy. In addition to the cosmetic products found in our investigation, here are also several patented cosmetic formulations for sensitive skin containing palmitoyl tripeptide-8 [40,41,42,43].

#### 3.2.3. Acetyl Tetrapeptide-15

**Scheme 3:**
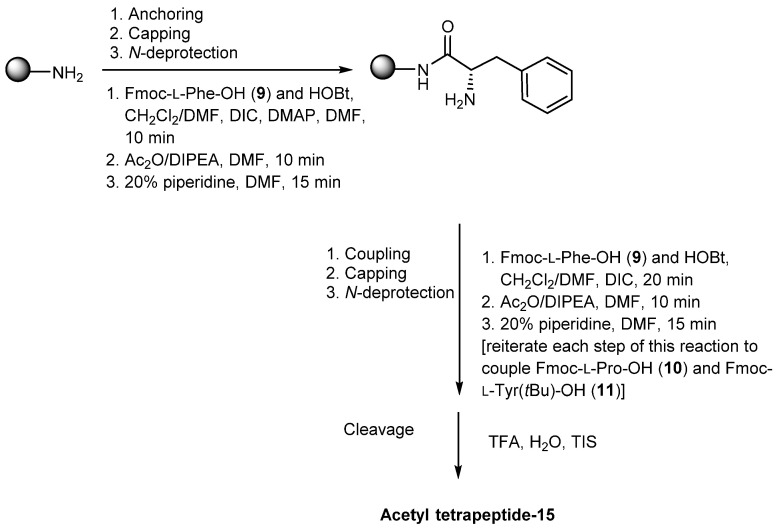
Solid-phase synthesis of acetyl tetrapeptide-15. HOBt: hydroxybenzotriazole; DIC: *N,N*′-diisopropylcarbodiimide; DMAP: 4-(dimethylamino)pyridine; DIPEA: *N,N*-diisopropylethylamine. Reproduced from reference with permission from the Centre National de la Recherche Scientifique (CNRS) and the Royal Society of Chemistry [46].

Deriving from endomorphin-2 (Tyr-Pro-Phe-Phe-NH_2_), a human μ-opioid agonist with selective anti-nociceptive effect, acetyl tetrapeptide-15 is a synthetic peptide constituted by the sequence *N*-acetyl-L-tyrosyl-L-prolyl-L-phenylalanyl-L-phenylalaninamide (Figure 1) [44,45]. Although this peptide is widely used in skincare formulations for sensitive skin, its synthesis is not fully described. However, the synthesis of a novel biologically active compound, the conjugate of jasmonic acid and of acetyl tetrapeptide-15, discloses that the synthesis of this tetrapeptide proceeds via a solid-phase method using AM RAM resin and the Fmoc/But procedure (Scheme 3) [46]. After initial treatment of the resin, the synthesis of the tetrapeptide proceeds with the addition of the resin to a mixture of hydroxybenzotriazole (HOBt) and Fmoc-L-Phe-OH (**9**). The reaction proceeds with anchoring of the Fmoc-L-Phe-OH (**9**) to the Rink amide resin (RAM) followed by protection of unreacted hydroxyl groups of the resin by capping, and deprotection of the Fmoc group. Further addition of protected amino acids Fmoc-L-Phe-OH (**9**), Fmoc-L-Pro-OH (**10**), Fmoc-L-Tyr(tBu)-OH (**11**) to the obtained amide, capping, *N*-Fmoc deprotection, and cleavage from the resin allows to obtain acetyl tetrapeptide-15 [46].

Acetyl tetrapeptide-15 was developed with the aim to reduce skin hyperreactivity producing inflammatory, chronic and neuropathic pain, by increasing the threshold of neuronal excitability in μ-opioid receptor via an endorphin-like pathway [47,48]. The efficacy of this peptide was demonstrated both in vitro and in vivo by the supplier [44]. Firstly, acetyl tetrapeptide-15 was tested regarding its ability to modulate the release of CGRP. CGRP is released after the activation of TRVP1 by capsaicin, heat, or depolarizing agents such cations [45]. The test was performed by incubating sensory neurons with acetyl tetrapeptide-15 (0.0003% and 0.001%), capsazepine (10 μM, a TRPV1 antagonist), or verapamil (100 μM, a calcium channel blocker) for 6h, which were then exposed to KCl and capsaicin. Acetyl tetrapeptide-15 0.001% reduced CGRP release very significantly, both when neurons were exposed to capsaicin and KCl, performing better than capsazepine 10 μM and similarly to verapamil 100 μM. The ability of acetyl tetrapeptide-15 to activate μ-opioid receptors from cultured sensory neurons in a capsaicin media was evaluated in competition with naloxone, a receptor antagonist. Capsaicin binds to TRVP1 receptors, thus eliciting a calcium influx through the cell membrane that produces CGRP, as well as a nervous influx signaling pain and discomfort. The presence of acetyl tetrapeptide-15 significantly reduced the CGRP release by capsaicin-stimulated neurons, but this effect was compromised in the presence of naloxone, reinforcing that acetyl tetrapeptide-15 binds to μ-opioid receptors. The activation from μ-opioid receptors inhibits the TRVP1 response by reducing the phosphorylation of adenylate cyclase (ADC) to protein kinase A (PKA). Lastly, a split-faced single-blind clinical study elucidated the ability of acetyl tetrapeptide-15 to reduce skin sensitivity after the exposure to capsaicin in 20 individuals. The protocol started with the application of increasing concentrations of a capsaicin solution in the nasolabial folds, to determine the concentration, which induced discomfort. A vehicle solution was applied to the other side of the face. Then, a 0.0015% solution with acetyl tetrapeptide-15 was applied twice daily, for four days, and the application of increasing capsaicin concentrations was repeated. Overall, there was a significantly increase in the capsaicin threshold which provoked discomfort in volunteers.

There is a patent referring to the use of acetyl tetrapeptide-15 in a cosmetic product for sensitive skin [45], but no studies were found in scientific literature for this compound.

#### 3.2.4. Palmitoyl Tripeptide-5

**Scheme 4:**
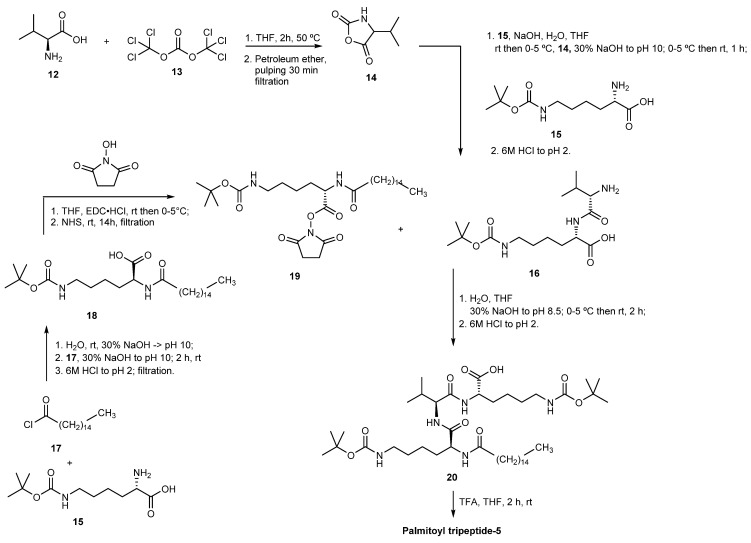
Liquid-phase synthesis of palmitoyl tripeptide-5. EDC: 1-ethyl-3-(3-dimethylaminopropyl)carbodiimide. Adapted from [50].

Palmitoyl tripeptide-5 is a fragment of Thrombospondin I (TSP-1) presenting the sequence, *N*-(1-oxohexadecyl)-L-lysyl-L-valyl-L-lysine (Figure 1) [21,49]. Two different liquid-phase methodologies were described for the synthesis of this tripeptide [50,51], designed to surpass some of the disadvantages associated with the solid-phase synthetic methodologies (high costs and pollution to the environment) [52]. One methodology (Scheme 4) involves a convergent synthesis with the initial formation of a *N*-carboxyanhydride **14** by reaction of L-valine (**12**) with phosgene (**13**) [50]. Boc-L-lysine (**15**) is then coupled to the *N*-carboxyanhydride **14**, forming Boc-protected dipeptide **16**. In a convergent route, *N*-acylated aminoacid **18** is prepared from Boc-L-lysine (**15**) and palmitoyl chloride (**17**). EDC/NHS Activation of the carboxyl group of Pal-Lys(Boc)-OH (**18**) produces Pal-Lys(Boc)-OSu (**19**). Coupling of intermediates **16** and **19** and further Boc deprotection furnishes palmitoyl tripeptide-5 [50].

**Scheme 5:**
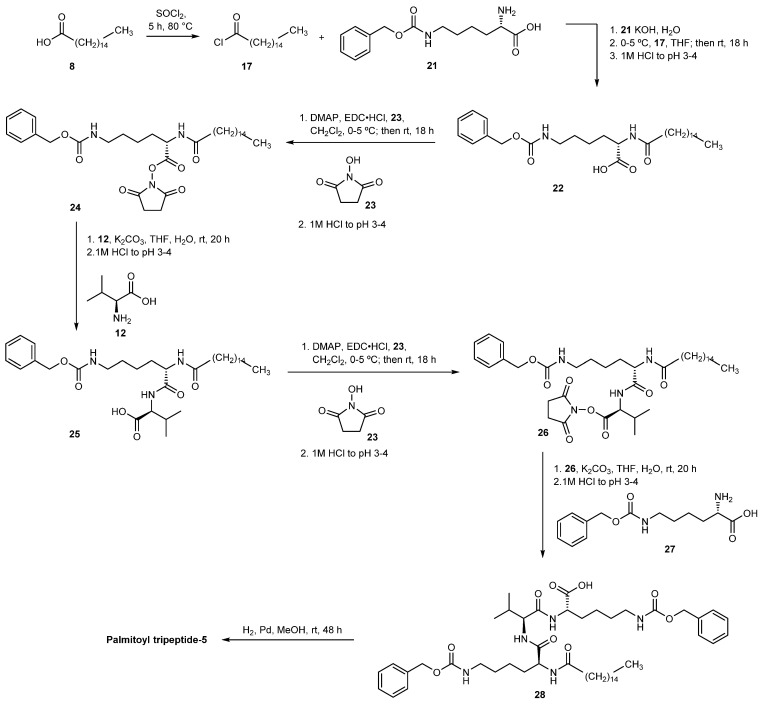
Liquid-phase synthesis of palmitoyl tripeptide-5. Adapted from [51].

The other synthetic methodology reported for the preparation of palmitoyl tripeptide-5 [51], although via a linear strategy (Scheme 5), is quite similar to the depicted in Scheme 4. Initial formation of palmitoyl chloride (**17**) from palmitic acid **8**, followed by coupling with benzyloxycarbonyl (Cbz)-L-lysine **21** forms Pal-Lys(Cbz)-OH (**22**) [51]. Further activation of the carboxyl group of Pal-Lys(Cbz)-OH (**22**) with NHS (**23**) and coupling with L-valine (**12**), followed by a second activation of the carboxyl group of **25** with NHS (**23**) and coupling with Cbz-L-lysine (**27**) furnishes Pal-Lys (Cbz)-Val-Lys(Cbz)-OH (**28**). Final deprotection of the Cbz groups allows to obtain palmitoyl tripeptide-5 [51].

Palmitoyl tripeptide-5 was used in a cosmetic ingredient mix from a raw material supplier, also containing spent grain wax and conjugated linoleic acid (CLA), for reducing skin redness in type I rosacea [53]. This ingredient mix is currently unavailable. Palmitoyl tripeptide-5 is proposed to reduce metalloproteases (MMP’s) expression and pro-inflammatory cytokine syntheses, causing vasodilation and capillary permeability [54]. However, neither efficacy studies for the use of palmitoyl tripeptide-5 alone or in this mix in rosacea of sensitive skin are available. This peptide has also been used in patented cosmetic formulations for sensitive skin [55,56,57]. Palmitoyl tripeptide-5 is also used in anti-aging cosmetic products, due to its ability to reduce MMP’S and promote the synthesis of type I and type II collagen from extracellular matrix, as well as for inhibiting melanin production by reducing tyrosinase activity [51,58].

#### 3.2.5. Acetyl Hexapeptide-49

Although acetyl hexapeptide-49 is widely used in the cosmetic industry, neither the structure nor the synthesis was reported to date in the literature.

This compound aims to regulate proteinase activated receptor 2 (PAR-2) from mast cells by trypsin-like serine proteases, thus reducing the inflammatory response which leads to IL-6 and IL-8 production, as well as TRVP-1 activation and subsequent CGRP release [59,60]. The supplier presents three studies for elucidating acetyl hexapeptide-49 efficacy. Primary human epidermal keratinocytes were incubated with vehicle or increasing concentrations of an acetyl hexapeptide-49 solution, and then exposed to 50 μM PAR-2 agonist. Cytokine production was determined by an ELISA test. At 0.5 mg/mL acetyl hexapeptide-49, there was a 69.6% and 71.5% decrease in IL-6 and IL-8 production, respectively. Moreover, cicatrization and barrier function recovery assays were performed using the same in vitro model. In this regard, keratinocytes treated with acetyl hexapeptide-49 solution were subject to an injury (cell-free area) induced by scraping a monolayer with a pipette tip, and then a cell proliferation assay was performed through the enzymatic conversion of the non-fluorescent calcein. The barrier function was recovered in both essays (concentrations are not disclosed). Another study in a reconstructed epidermis model was performed for evaluating the ability of a 4% acetyl hexapeptide-49 solution for reducing response to cosmetic allergens. The skin model was exposed both to hexyl cinnamal and farnesol, allergens, for 24 h, and the IL-8 expression was determined by an ELISA test. The 4% acetyl hexapeptide-49 allowed to reduce IL-8 expression in 58.2% comparing to positive control. Noteworthy, the skin model used in this study is not revealed, which would be important to evaluate its susceptibility to these allergens. Additionally, a clinical study was performed using 25 volunteers (24 to 67 years) who were selected based on their lactic acid stinging susceptibility. At the beginning of the study, volunteers applied a 10% lactic acid solution on the nasolabial fold, followed by a cream containing 2% acetyl hexapeptide-49. The soothing effect was evaluated after one hour, and volunteers reported an improvement in the stinging sensation. Then, the cream was applied twice a day for 7 days, and once again, the stinging sensation was assessed. After this period, a 32% reduction in volunteers experiencing stinging was found. Lastly, the supplier reported another clinical study including 20 volunteers (18 to 55 years) who applied a cream containing 2% acetyl hexapeptide-49 on the left leg, and a vehicle formulation on right leg twice a day for four weeks. Skin moisturization was evaluated by corneometry, and a clinical assessment of skin dryness, scaling, smoothness, softness, and suppleness was performed by a dermatologist. After four weeks, the supplier reported a significant increase in skin hydration comparing to vehicle, and the skin appeared less dry and scaly, smoother, softer, and more supple. Two patents for cosmetics with acetyl hexapeptide-49 have also been found [60,61].

#### 3.2.6. Palmitoyl Tetrapeptide-7

**Scheme 6:**
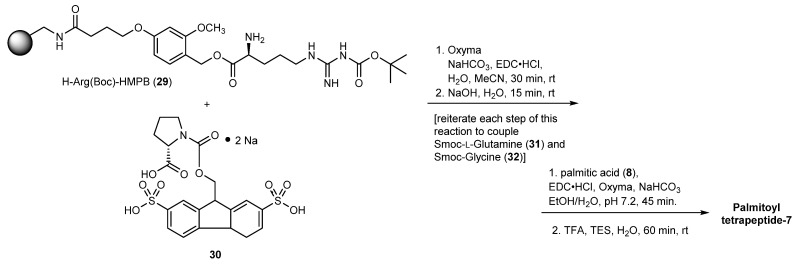
Solid-phase synthesis of palmitoyl tetrapeptide-7. Adapted from [63].

Palmitoyl tetrapeptide-7 is a fragment of immunoglobulin G presenting the sequence *N*-(1-oxohexadecyl)glycyl-L-glutaminyl-L-prolyl-L-arginine (Figure 1) [62]. Two different solid-phase methodologies were described for the synthesis of this tetrapeptide [63,64]. In the first methodology (Scheme 6), a preloaded H-Arg(Boc)-HMPB-ChemMatrix resin (**29**) (functionalized support acylated with Riniker’s super-acid-sensitive (4-hydroxymethyl-3-methoxyphenoxy)butanoic acid handle) is used and the first amino acid is attached by coupling with 2,7-disulfo-9-fluorenylmethoxycarbonyl (Smoc)-proline sodium salt (**30**). After resin wash and Smoc deprotection, coupling of the next amino acid is performed (Smoc-glutamine (**31**), and a solution of Smoc-glycine (**32**)), which was used without side-chain protecting group) until the desired peptide is completed. Oxyma [ethyl 2-cyano-2-(hydroxyimino)acetate] is an additive in the coupling medium safer than benzotriazole-based additives such as HOBt. Palmitoylation and cleavage of the peptide from the resin followed by precipitation and lyophilization gives the desired palmitoyl tetrapeptide-7 [63].

The second methodology involves the presence of a soluble fragment to improve the water solubility of the palmitoyl tetrapeptide-7, so that it is easier to purify [64]. Hence, five hydrophilic lysines are continuously coupled on the amino resin, then a connecting arm of *p*-hydroxybenzoic acid is introduced, and finally the remaining amino acids are coupled according to the peptide sequence of the palmitoyl tetrapeptide-7 (Pal-Gly-Gln-Pro-Arg-OH) [64]. HOBt/DIC methodology is adopted as a coupling approach when other amino acid residues (except the first lysine) are coupled [64]. Removal of the Fmoc protecting groups, resin cleavage, and further purification gives the refined peptide which is hydrolyzed to the target peptide palmitoyl tetrapeptide-7 [64].

Palmitoyl tetrapeptide-7 decreases IL-6 secretion, reduces inflammation after UVB exposure and stimulates laminin IV and V as well as collagen VII production [62]. In this regard, palmitoyl tetrapeptide-7 has been used in anti-aging cosmetics [65,66]. Although this mechanism of action is promising in the regulation of skin inflammation, namely for wound healing, no studies were found revealing palmitoyl tetrapeptide-7′s efficacy in this regard, nor for controlling the symptoms of sensitive skin [67]. Two patents however describe its use in cosmetic products for sensitive skin [68,69].

#### 3.2.7. Palmitoyl Oligopeptide

The name “palmitoyl oligopeptide” was “removed” in 2013, since was used to designate two distinct molecules from the time of its development in 1994. The two compounds were renamed as palmitoyl tripeptide-1 (Pal-GHK) and palmitoyl hexapeptide-12 (Pal-KTTKS) in order to clarify the composition of cosmetic products [70].

**Scheme 7:**
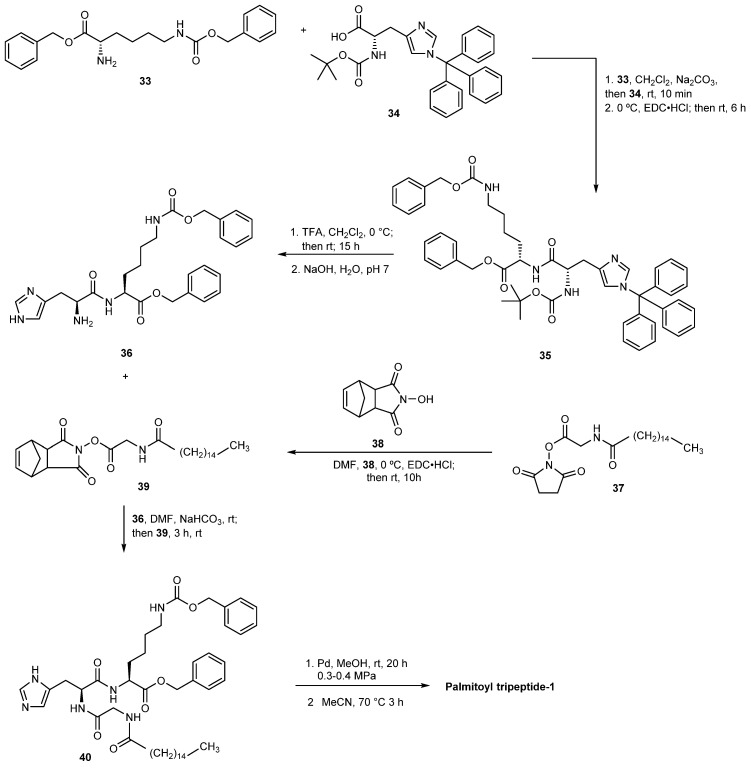
Liquid-phase synthesis of palmitoyl tripeptide-1. NHS: *N*-Hydroxysuccinimide. Adapted from [73].

Palmitoyl tripeptide-1 is a collagen fragment presenting the sequence *N*-(1-oxohexadecyl)glycyl-L-histidyl-L-lysine (Figure 1) [71]. To date, three different methodologies were reported for the synthesis of this tripeptide [63,72,73]. The first methodology (Scheme 7) [73] consists in an initial EDC-mediated coupling of H-Lys(Z)-OBzl×HCl (**33**) and Boc-His trityl(Trt)-OH (**34**) followed by removal of the trityl and Boc protecting groups affords H-His-Lys (Z)-OBzl (**36**). Coupling of this dipeptide **36** with Pal-Gly-ONb (**39**), previously synthesized from Pal-Gly-OSu (**37**) gives Pal-Gly-His-Lys (Z)-OBzl (**40**) which, after removal of Cbz and Bzl protecting groups affords palmitoyl tripeptide-1 [73].

Another reported methodology for the synthesis of this tripeptide is performed by using the same methodology as used for the synthesis of palmitoyl tetrapeptide-7 (Scheme 6). A preloaded H-Lys(Boc)-HMPB-ChemMatrix resin to which were coupled Smoc-L-Hys and Smoc-Gly is used in a solid-phase approach. Deprotection of the Smoc protecting group, palmitoylation, cleavage of the peptide from the solid support, and further lyophilization affords palmitoyl tripeptide-1 [63].

**Scheme 8:**
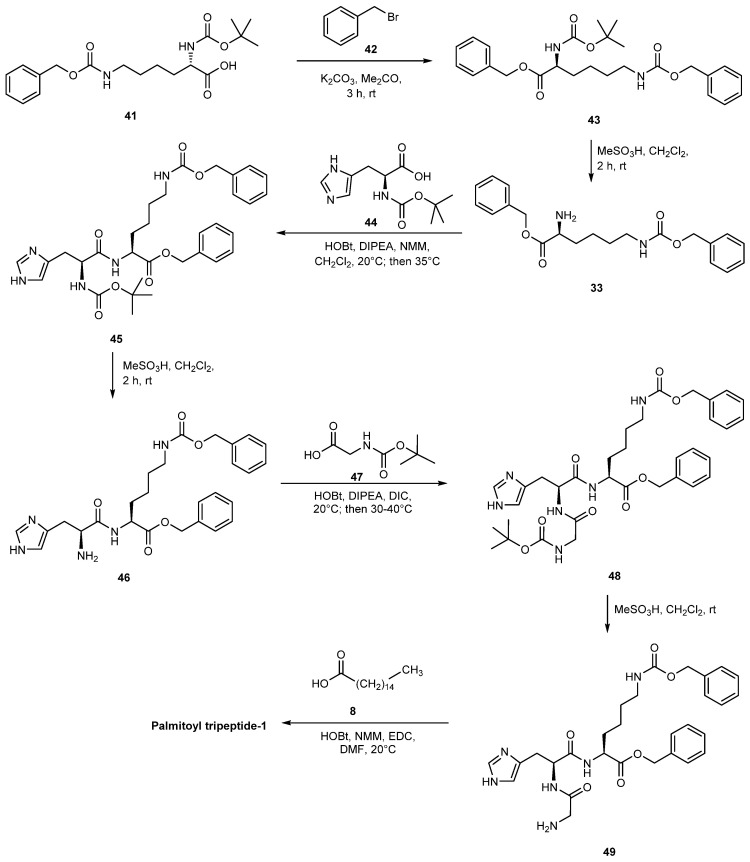
Liquid-phase synthesis of palmitoyl tripeptide-1. HOBt: hydroxybenzotriazole; DIPEA: *N,N*-diisopropylethylamine; *N*-methylmorpholine; DIC: *N,N*′-diisopropylcarbodiimide; EDC: 1-ethyl-3-carbodiimide hydrochloride. Adapted from [72].

The third methodology is initiated through the protection of the carboxylic moiety of Boc-Lys-(Z)-OH (**41**) to obtain Boc-Lys(Z)-OBzl (**43**) (Scheme 8) [72]. Boc removal in acidic conditions, followed by coupling with Boc-His-OH (**44**), under usual coupling conditions (HOBt, NMM, DIPEA) gives Boc-His-Lys(Z)-OBzl (**45**) which, after deprotection/coupling with Boc-Gly-OH (**47**) and deprotection/palmitoylation with palmitic acid (**8**), and final deprotection of the carboxylic moiety with Pd/C furnishes the desired palmitoyl tripeptide-1.

**Scheme 9:**
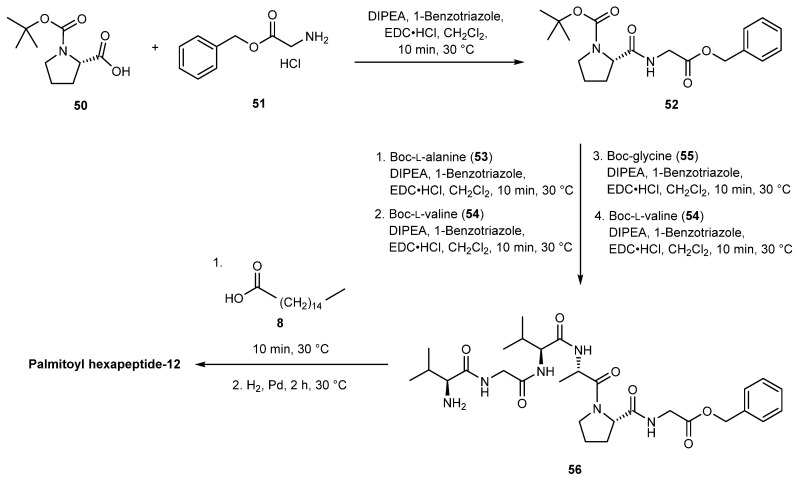
Synthesis of palmitoyl hexapeptide-12. Adapted from [74].

Palmitoyl hexapeptide-12 is an elastin fragment presenting the sequence *N*-(1-oxohexadecyl)-L-valyl-glycyl-L-valyl-L-alanyl-L-prolyl-glycine (Figure 1). The methodology for the preparation of this peptide is similar to the previously reported for other liquid-phase peptide syntheses, consisting in a series of deprotection/coupling reactions starting with the initial coupling of Boc-L-proline (**50**) and benzyl glycinate (**51**), followed by the coupling of Boc-L-alanine (**53**), Boc-L-valine (**54**), Boc-glycine (**55**), and Boc-L-valine (**54**) (Scheme 9) [74].

Although both peptides have been used in anti-aging cosmetics [22], neither studies or patents revealing the use of palmitoyl oligopeptide, palmitoyl tripeptide-1, or palmitoyl hexapeptide-12 for reducing sensitive skin symptoms were found.

#### 3.2.8. Highlights in the Usage of Synthetic Peptides in Cosmetics for Sensitive Skin

Peptides disclosed in the composition of the facial cosmetics for sensitive skin investigated are all from synthetic origin. In contrast to natural peptides that have variable dimensions, reaching high molecular weights, and may contain allergenic moieties and their extraction can be costly [22], the synthesis of simpler peptides has the advantage of reaching both the pharmacophoric portion and improved bioavailability. Most peptides found in cosmetic products for sensitive skin are based on the pharmacologically active portions of endogenous molecules, whose low molecular weight and subsequent hydrophobization provide a better penetration through the stratum corneum. Noteworthy, acetyl dipeptide-1 cetyl ester, palmitoyl tripeptide-8, and acetyl tetrapeptide-15 are neurotransmitter-inhibiting peptides acting as agonists from cutaneous opioid system, such as µ receptor, which interacts with TRVP1 receptors through intracellular signaling. Therefore, these peptides reduce the activation of cutaneous nerve fibers, especially through TRVP1, thus preventing the release from CGRP. Conversely, acetyl hexapeptide-49 also reduces CGRP release after TRVP1 activation as well as the pro-inflammatory cytokine production by a signaling pathway involving proteinase activated receptor 2 (PAR-2). Interestingly, no peptide acting directly on TRVP1 receptors has been found. There are several TRVP1 antagonists reported in the scientific literature, but no peptides have been described, possibly due to the specificity of the receptor’s binding site [75].

Concerning synthetic methodologies, these usually proceed via a linear approach, although a few convergent approaches were also described. Additionally, the fine-tuning of the chemistry associated with the synthetic methodology, ranging from standard coupling procedures (EDC/NHS) to new and improved methodologies, such as greener methods (EDC/Oxyma) can also contribute for the marked increase of peptides in this industry. The main bottleneck of these procedures is related to the activation of one of the carboxylic groups before the occurrence of the coupling reaction. This activation step, along with the next coupling reaction can lead to a potential loss of chiral integrity at the carboxyl residue undergoing activation. Although the above-described methodologies for the preparation of these peptides are fully optimized, the development of new procedures might need to take this challenge into account. New stand-alone coupling reagents, such as HOAt, containing better leaving groups can be used to enhance coupling rates and reduce the risk of racemization. Oxyma, a highly efficient leaving group, is safer and less hazardous than HOAt. Oxyma exhibited the same efficiency as HOAt and greater performance than HOBt. Of similar importance, is also the use of protecting groups, which can be used to maximize the yield of the desired products, as well as to minimize undesirable side reactions such as polymerization of the amino acids, usual in the synthesis of complex peptide-based structures.

### 3.3. Applicability of the Described Synthetic Peptides in Pharmeceuticals

Synthetic peptides whose mechanism of action has been previously act indirectly on TRPs. These receptors are associated with several skin diseases [76]. Pruritus, also known as itch, can be idiopathic or secondary to different pathologies, such as atopic dermatitis, psoriasis, urticaria, chronic renal failure, or liver diseases [77]. TRP channels, namely transient receptor potential cation channel subfamily A member 1 (TRPA1) and TRPV1 have shown to be greatly involved in itch development both under both physiological and pathological conditions. Rosacea is also aggravated by neuroinflammation, and the overexpression of TRPV1, TRPV2, TRPV4, and TRPV4 receptors has been proved in distinct subtypes from the disease [78]. Palmitoyl tripeptide-8, which is present in the formulation of a cosmetic product with proven efficacy for the treatment of rosacea patients, may be a prime candidate for the development of pharmaceuticals aimed at alleviating the signs and symptoms of this condition [39]. Moreover, the overexpression of TRPV1, TRPV4, and TRPV6 has been associated to nonmelanoma skin cancer, but their carcinogenic effect remains unknown [76].

Therefore, the recognition of the effectiveness from these synthetic and the further improvement of their molecular structures, may be useful for modulating TRP associated pathways, providing alternative treatments and/or symptom management for multiple diseases. These compounds are known to be safe for topical application, and their toxicity has been assessed both by the manufacturer, through material safety data sheets, and by the independent committee Cosmetic Ingredient Review supporting the Federal drug Administration in the US [79].

## 4. Conclusions

This study characterizes the usage of peptides in cosmetic products for sensitive skin for the first time. These ingredients were present in about 17% of the facial cosmetics for sensitive skin analyzed in 2019. Seven distinct peptides were found, namely acetyl dipeptide-1 cetyl ester, palmitoyl tripeptide-8, acetyl tetrapeptide-15, palmitoyl tripeptide-5, and acetyl hexapeptide-49, for which experimental data is reported to support use in cosmetics for sensitive skin, along with palmitoyl terrapeptide-7 and palmitoyl oligopeptide (the old name for the peptides palmitoyl tripeptide-1 and palmitoyl hexapeptide-12), whose efficacy is only documented for anti-aging cosmetics. Most of the available information regarding these ingredients is not reported in peer-reviewed scientific journals, but rather in patents and supplier brochures. Additionally, the small number of randomized clinical studies, and especially the fact that only one study included volunteers with sensitive skin (acetyl dipeptide-1 cetyl ester), hinders a robust evidence of the in vivo efficacy of these peptides. More clinical studies with good methodological quality are needed to provide sound evidence of the peptide’s efficacy in sensitive skin care.

From a chemical perspective, the increasingly use of these peptides as ingredients in the cosmetic industry can be explained as a result of the development of solid-phase syntheses and automated methodologies. Finally, the implementation of reverse-phase high-performance liquid chromatography for peptide purification, in combination with the previously mentioned factors, has allowed the production of complex peptides in multi-kilogram amounts that was impossible to envisage only a few decades ago and that contributed to a boost in the use of peptides as ingredients in cosmetic formulations.

Peptides are useful ingredients in cosmetics for sensitive skin that may also be relevant for medical devices or medicines intended to treat or prevent the symptoms of diseases in which neurogenic inflammation plays an important role, such as rosacea and atopic or seborrheic dermatitis. Given the worldwide prevalence of sensitive skin and the growing interest in peptides by the cosmetic industry, it is foreseeable that the market for these products may increase in the coming years, fostering the design of new and more effective compounds. In the future, it is possible to see a further exploration of signaling pathways involving cutaneous opioid receptors, through the development of peptides acting upstream in this pathway, or as agonists of opioid receptors. Moreover, the development of peptides acting directly on TRVP1 receptors, either extra or intracellularly, could provide promising results.

## Figures and Tables

**Table 1:** Peptides found at INCI lists of cosmetic products for sensitive skin and their relative usage (%).

INCI	Classification	Relative Usage (%)
Acetyl Dipeptide-1 Cetyl Ester	Neurotransmitter-inhibiting	5.7
Palmitoyl Tripeptide-8	Neurotransmitter-inhibiting	4.5
Acetyl Tetrapeptide-15	Neurotransmitter-inhibiting	2.3
Palmitoyl Tripeptide-5	Signal	2.3
Acetyl Hexapeptide-49	Unknown	1.1
Palmitoyl Tetrapeptide-7	Signal	1.1
Palmitoyl Oligopeptide	Signal	1.1

## Data Availability

Data sharing not applicable.

## Limitations

This study was performed for the Portuguese cosmetic market, which is dominated by multinational cosmetic brands. Therefore, this may result in discrepancies when comparing with other markets. Many ingredients found in cosmetic products from the market lack scientific literature regarding their efficacy. Therefore, some of the information used in this study was collected in technical documents and patents from suppliers.

## Abbreviations and Acronyms

α-MSH	α-melanocyte stimulating hormone
ACTH	adrenocorticotropin
ADC	adenylate cyclase
AQP3	aquaporin 3
Boc	*tert*-Butyloxycarbonyl
Bu	Butyl
Bz	benzoyl
Cbz	benzyloxycarbonyl
CLA	conjugated linoleic acid
DCC	*N*,*N*’-dicyclohexylcarbodiimide
DIC	*N*,*N*′-diisopropylcarbodiimide
DIPEA	*N*,*N*-diisopropylethylamineDMAP
DMAP	4-(dimethylamino)pyridine
DMF	*N*,*N*-dimethylformamide
EDC	1-ethyl-3-(3-dimethylaminopropyl)carbodiimide
FLG	filaggrin
Fmoc	fluorenylmethyloxycarbonyl
h	hours
HBTU	*N*,*N*,*N*′,*N*′-tetramethyl-*O*-(1*H*-benzotriazol-1-yl)uronium hexafluorophosphate
HOBt	hydroxybenzotriazole
INCI	international nomenclature of cosmetic ingredients
MMP’s	metalloproteases
MSHs	melanocyte-stimulating hormones
NF-κβ	nuclear factor κ-β
NHS	*N*-hydroxysuccinimide
NMP	*N*-methyl-2-pyrrolidone
Pal	Palmitic acid
PAR-2	proteinase activated receptor 2
PKA	protein kinase A
POMC	pro-opiomelanocortin
PTSA	*p*-toluenesulfonic acid
RAM	Rink amide
rt	room temperature
SDS	sodium dodecyl sulfate
Smoc	2,7-disulfo-9-fluorenylmethoxycarbonyl
Su	succinimide
TEA	triethylamine
TFA	trifluoroacetic acid
THF	tetrahydrofuran
TIS	triisopropylsilane
Trt	Trityl
TRPV	Transient Receptor Potential Cation Channel Subfamily V
TPPA	Transient Receptor Potential Cation Channel Subfamily A
TSP-1	thrombospondin I
